# Thrombocytes Correlate with Lymphangiogenesis in Human Esophageal Cancer and Mediate Growth of Lymphatic Endothelial Cells *In Vitro*

**DOI:** 10.1371/journal.pone.0066941

**Published:** 2013-06-26

**Authors:** Sebastian F. Schoppmann, Lejla Alidzanovic, Andrea Schultheis, Thomas Perkmann, Christine Brostjan, Peter Birner

**Affiliations:** 1 Department of Surgery, Medical University of Vienna, Vienna, Austria; 2 Department of Pathology, Hospital Rudolfsstiftung, Vienna, Vienna, Austria; 3 Department of Laboratory Medicine, Medical University of Vienna, Vienna, Austria; 4 Department of Pathology, Medical University of Vienna, Vienna, Austria; 5 Comprehensive Cancer Center, Gastroesophageal Unit, Medical University of Vienna, Vienna, Austria; Vanderbilt University, United States of America

## Abstract

Recent data provide evidence for an important role of thrombocytes in lymphangiogenesis within human malignant disease. The aim of this study was to investigate the role of thrombocytes in lymphangiogenesis in human esophageal cancer. Perioperative peripheral blood platelet counts (PBPC) were evaluated retrospectively in 320 patients with esophageal cancer, comprising 184 adenocarcinomas (AC), and 136 squamous cell carcinomas (SCC). Data on lymphangiogenesis evaluated by anti-podoplanin immunostaining were available from previous studies, platelets within the tumor tissue were assessed by CD61 immunostaining. For *in vitro* studies, human lymphatic endothelial cells (LECs) were isolated and co-cultured with peripheral blood platelets. Stromal thrombocytic clusters (STC) were evident in 82 samples (25.6%), and vascular thrombocytic clusters (VTC) in 56 (17.5%). STC and VTC were associated with a significantly higher PBPC at investigation of all cases. The presence of STC was associated with higher lymphatic microvessel density (p<0.001), PBPC and STC were associated with lymphovascular invasion of tumor cells in a regression model. The presence of STCs was associated with shorter DFS of all patients (p = 0.036, Breslow test), and VTC with shorter DFS in in SCC (p = 0.025, Breslow test). In cell culture, LEC proliferation was enhanced by co-culture with human platelets in a dose- and time-dependent manner mediated by the release of PDGF-BB and VEGF-C. Platelets play an important role in lymphangiogenesis and lymphovascular invasion in esophageal cancer, influencing prognosis. So the disruption of signaling pathways between platelets, tumor cells and lymphatic endothelium might be of benefit for patients.

## Introduction

Esophageal adenocarcinomas (AC) of the gastroesophageal junction are believed to be mainly induced by gastroesophageal reflux, while squamous cell carcinomas (SCC) are mainly attributed to alcohol and tobacco consumption [1], [2]. In the last decades, the incidence of AC has raised dramatically in western countries [3]. Despite multimodal therapeutic strategies, overall outcome for patients with gastroesophageal cancer remains poor. So novel therapeutic concepts are urgently needed, and insights into the pathophysiology of disease are a prerequisite for their development. Like many other carcinomas, esophageal cancers mainly spread through the lymphatic system, and lymphovascular invasion of tumor cells is associated with diminished prognosis of patients [4].

Today striking evidence exists that tumors may establish not only their own blood vessels supply, but might also induce the formation of new lymphatic vessels (lymphangiogenesis) and to migrate actively into this newly formed vessels to promote their spread [5].

So possible inhibition of this process might be of benefit for patients, especially as recent data suggest that the process of lymphangiogenesis and lymphatic vessel invasion (LVI) is not only limited to primary tumors, but is also evident in lymph node metastases, resulting in further spread of tumor cells [6].

The formation of tumor associated lymphatic vessels is a complex process and currently only partially understood. During embryonic angiogenesis, platelets seem to play a key role in separating blood and lymphatic vasculature [7]. But there is increasing evidence that platelets might interact with the human lymphatic system also in malignant disease, and might facilitate lymphangiogenesis and tumor metastasis [8], [9].

The aim of this study was to investigate the role of platelets with regard to lymphangiogenesis in human esophageal cancer.

## Materials and Methods

### Patients

All patients who underwent surgical resection of carcinomas of the esophagus or the gastroesophageal junction between 1992 and 2011 at the Department of Surgery, Medical University of Vienna, were included into this study if sufficient tissue and preoperative thrombocytic count were available. Tumors of all patients were re-evaluated according to the UICC 7th edition TNM staging.

### Ethics Statement

Institutional review board approval was obtained (Institutional Review Board of the Medical University of Vienna, Austria, EK 1122/2009). Due to the retrospective nature of this study, using only archived tissue, no informed consent of patients was required and obtained, as approved by the review board. The specific samples used in this study have already been used in previous publications [4], [10]–[15].

### Analysis of Peripheral Blood Platelet Count (PBPC)

PBPC of patients was determined routinely before surgery and also before initiation of neoadjuvant chemotherapy, if administered.

Analysis of PBPC was routinely performed on standard automated hematology analyzers at the Department of Laboratory Medicine, Medical University of Vienna. During the observational period from 1992 to 2011 the following hematology analyzers were applied: until 1995 Coulter STKS (Coulter, Hialeah, FL), from 1995 to 2001 Sysmex NE-8000 (TOA Medical Electronics, Kobe, Japan), since 2001 Sysmex XE-2100 (Sysmex Corporation, Kobe, Japan).

### Immunostaining

Immunohistochemistry (IHC) was performed on paraffin-embedded specimens fixed in 4% buffered formalin, using three µm thick histological sections.

Data on lymphatic vessels assessed by the monoclonal mouse anti-podoplanin antibody D2-40 (Ventana Medical Systems, Tucson, Arizona) were available from previous studies [4], [15].

For detection of thrombocytes immunostaining was performed using a monoclonal anti CD61 antibody (NCL-CD61-308, Leica Biosystems, Newcastle, UK) in a dilution of 1∶1600. A Benchmark Ultra immunostainer (Ventana Medical Systems, Tucson, Arizona) was used for immunohistochemistry.

Analysis of anti-podoplanin immunostaining was performed as described previously [16]:

In brief, for determination of LMVD, the area within or directly adjacent to tumor formations with the greatest number of distinctly highlighted microvessels (“hot spot”) was selected at low magnification. LMVD was then determined by counting all immunostained vessels at a total magnification of x200 in an examination area of 0.25 mm^2^. A case was considered as positive with regard to LVI when at scanning of the whole immunostained slide a tumor cell cluster was visible within a podoplanin decorated vascular space.

**Figure 1:**
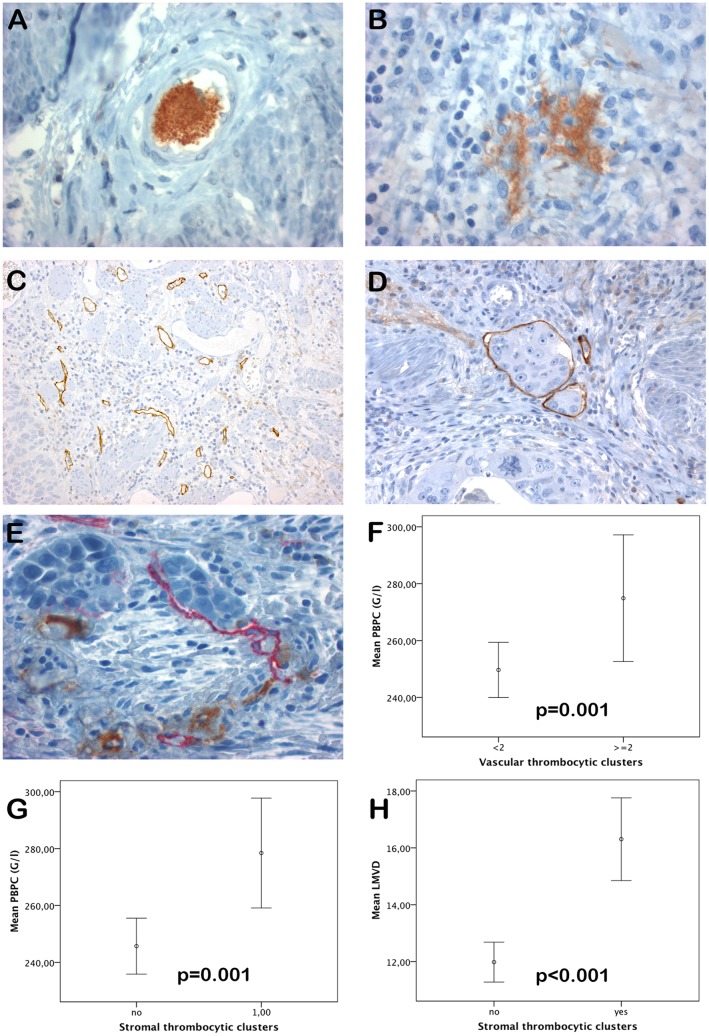
Samples and results of immunohistochemistry. A: Vascular thrombocytic cluster (VTC) in an esophageal cancer specimen (original magnification x400). B: Stromal thrombocytic cluster (STC) in an esophageal cancer specimen assessed by anti – CD61 immunostaining (original magnification x400). C: Esophageal cancer specimen with high lymphatic microvessel density (LMVD) assessed by anti- podoplanin immunostaining (original magnification x200). D: Lymphovascular invasion of tumor cells assessed by anti-podoplanin immunostaining (original magnification x200). E: Double staining for lymphatic vessels using (red, anti-podoplanin) and thrombocytes (brown, anti- CD61) (original magnification x400). F–H: Error bars showing mean values±2× standard error. Peripheral blood platelet counts (PBPC) were significantly higher in samples with VTC (F). PBPC (G) and LMVD (H) were significantly higher in esophageal cancer samples with STC.

For analysis of anti-CD61 immunostaining, superficial, exulcerated or bleeding tumor areas were excluded from analysis. A tumor was scored as positive for thrombocytic clusters in vessels (VTC), if at least in two vessels such clusters were seen (Fig. 1A).

A tumor was considered as showing thrombocytic clusters within the tumor stroma (STC), if more than one unequivocal CD61 immunostained cluster was visible within the tumor stroma (Fig. 1B).

Analysis of immunohistochemistry was performed by two independent investigators (S.F.S., P.B.) blinded to clinical data. Cases with divergent results were evaluated together using a multiheaded microscope.

### Statistical Analysis

T-test, Mann-Whitney test, Chi square tests and linear regression were used as appropriate. All numbers given are mean values±standard deviations, if not stated otherwise.

Overall survival (OS) was defined as the time between primary surgery and the patient’s death, survival until the end of the observation period was considered as censored observation. Disease-free survival (DFS) was defined as time from the day of surgery until first evidence of disease-progression. Univariate analysis of survival was performed using Breslow test, multivariate analysis using the Cox proportional- hazards model. Patientś age, radicality of resection, tumor and lymph node stage (according to the current UICC classification), tumor grade and lymph node status were included into Cox regression. A two-tailed p-value of ≤0.05 was considered as significant, SPSS 19.0 was used for all calculations.

### Endothelial Cell Isolation and Culture

Primary endothelial cells were isolated from human foreskin samples by proteolytic digest, and purified using anti-CD31 antibody coupled magnetic beads (Invitrogen Corp., Carlsbad, CA). Isolates were cultured in microvascular endothelial growth medium EGM2-MV (Clonetics®, Lonza, Walkersville, MD) containing 1 µg/ml fibronectin, 5% FCS and human growth factors without the supplementation of vascular endothelial growth factor (VEGF). For further separation of lymphatic and blood endothelial cells (LECs and BECs), anti-podoplanin antibody coupled magnetic beads were applied. All isolates showed ≥98% purity and viability.

Cells were seeded at a density of 1×10^5^ in 30 mm wells for 24 h. After extensive washing with phosphate buffered saline, cells were incubated with 10^5^–10^7 ^gel filtered platelets in EBM-2 basic medium (Lonza) containing 0.5% FCS. For blocking experiments, the following reagents were added to co-cultures: goat anti-human PDGFR-β neutralizing IgG at 1 µg/ml (R&D Systems, Minneapolis, MN), mouse anti-human VEGFR-2 neutralizing IgG_1_ at 50 ng/ml (R&D Systems) and VEGFR-3/human Fc soluble competitor at 1 µg/ml (Cell Sciences, Canton, MA). For negative control, non-specific goat IgG, mouse IgG_1_ and human IgG were applied at the same concentrations.

Cell analysis was conducted after 24 to 72 hours. Digital images of cells were taken with an Axiovert 40CFL microscope (Zeiss, Oberkochen, Germany). Cells were subsequently released from culture plates by trypsinization and the cell count was assessed using trypan blue staining. Data shown represent mean and standard deviation of triplicate samples. Each experiment was conducted ≥3 times with LEC isolates from different donors.

### Platelet Isolation

Venous blood was drawn from healthy volunteers into sodium citrate tubes and subjected to centrifugation at 85×g and RT for 20 min. The obtained platelet-rich plasma supernatant was purified by gel filtration using sepharose 2B (Sigma-Aldrich, St. Louis, MO). Platelet activation during purification was inhibited with 100 µM prostaglandin E1. After centrifugation of gel-filtered platelets at 3000×g and RT for 1.5 min, platelets were resuspended in EBM-2 medium containing 0.5% FCS and the platelet concentration was determined with a Sysmex counter (Kobe, Japan).

### Formazan Based Cell Proliferation Assay

The non-radioactive cell proliferation and cytotoxicity assay (EZ4U®, Biomedica, Vienna, Austria) was used to determine the metabolic activity of LECs by tetrazolium reduction. 100 µl of dissolved chromogenic substrate were added to each 30 mm well and incubated at 37°C for 2 h. Thereafter, the culture supernatant was retrieved and the absorbance at 450 nm was measured with a Varioskan Flash plate reader (Thermo Fisher Scientific Inc., Waltham, MA).

### Growth Factor Measurements

Co-culture supernatants were analyzed for the content of VEGF-A, -C, -D and PDGF-BB by enzyme-linked immunosorbent assay (Quantikine; R&D Systems) according to manufacturer’s instructions.

## Results

### Surgical Specimens

In total, 320 invasive esophageal cancers were included into this study: 184 adenocarcinomas (AC), and 136 squamous cell carcinomas (SCC).

Clinical data of patients are compiled in table 1, neoadjuvant chemotherapy before surgery was administered in 98 patients. For calculations, in these patients generally PBPC before initiation of neoadjuvant chemotherapy were used. In 11 patients, no data on PBPC before neoadjuvant chemotherapy were available. Since no significant difference in the PBPC before and after neoadjuvant chemotherapy was found in the remaining 87 patients (p>0.05, t-test), PBPC after neoadjuvant chemotherapy (immediately before surgery) were used at these patients for analysis. STC were present in 82 samples (25.6%; 36 AC, 46 SCC), VTC in 56 (17.5%, 22 AC, 34 SCC). Figure 1 gives samples of immunostaining. Generally, STC (p = 0.004, Chi Square test) and VTC (p = 0.002, Chi square test) were more common in SCC compared to AC. A significant association between the presence VTCs and STCs was seen at investigation of all cases and at investigation of AC and SCC separately (p<0.001, respectively, Chi square test).

**Table 1:** Clinical data of patients and presence of stromal and vascular thrombocytic clusters.

Variable	Stromal thrombocytic clusters	Vascular thrombocytic clusters
**Adenocarcinoma**		
Tumor stage		
pT1a (n = 11)	0	0
pT1b (n = 18)	3 (16.3%)	1 (5.6%)
pT2 (n = 53)	11 (20.8%)	6 (11.3%)
pT3 (n = 93)	20 (21.5%)	14 (15.1%)
pT4 (n = 9)	2 (22.2%)	1 (11.1%)
Lymph node status (n = 173)		
pN0 (n = 57)	10 (17.5%)	5 (8.8%)
pN1 (n = 34)	3 (8.8%)	4 (11.8%)
pN2 (n = 35)	14 (40%)	6 (17.1%)
pN3 (n = 47)	8 (17%)	21 (12.1%)
Grading		
G1 (n = 6)	2 (33.3%)	1 (16.7%)
G2 (n = 73)	12 (16.4%)	8 (11%)
G3 (n = 105)	22 (21%)	12 (12.4%)
**Squamous cell cancer**		
Tumor stage		
pT1a (n = 7)	1 (14.3%)	1 (14.3%)
pT1b (n = 16)	3 (18.8%)	1 (6.3%)
pT2 (n = 33)	12 (36.4%)	8 (24.2%)
pT3 (n = 71)	28 (39.4%)	23 (32.4%)
pT4 (n = 9)	2 (22.2%)	1 (11.1%)
Lymph node status (n = 130)		
pN0 (n = 54)	14 (25.9%)	15 (27.8%)
pN1 (n = 46)	16 (34.8%)	10 (21.7%)
pN2 (n = 17)	7 (41.2%)	3 (17.6%)
pN3 (n = 13)	8 (61.5%)	5 (38.5%)
Grading		
G1 (n = 11)	4 (36.4%)	3 (27.3%)
G2 (n = 94)	36 (38.3%)	26 (27.7%)
G3 (n = 31)	6 (19.4%)	5 (16.1%)

While no association of the presence of STC with tumor staging and histological grading was seen in all cases and AC, in SCC more advanced lymph node status was seen in tumors with STC (median of pN1 in both cases, p = 0.024, Mann Whitney test).

The presence of VTC was associated with more advanced tumor stage in all cases (median of pT3 in both cases with a trend towards higher staging in patients with VTC, p = 0.036, Mann Whitney test), but this association was not seen when investigating AC and SCC separately.

PBPC were higher in cases with VTC at investigation of all cases (275±83 vs. 250±79 G/l, p = 0.001, t-test) (Fig. 1F), but this association was seen at separate investigation of tumor types only in SCC (282±75 vs. 243±81 G/l; p = 0.007, t-test) but not in AC (p = 0.077, t-test). No association of VTC with LMVD was seen in all cases and AC and SCC separately. (p>0.05, t- test).

The presence of STC was associated with a higher PBPC at investigation of all cases compared to patients without STC (279±88 G/l vs. 246±76 G/l; p = 0.001, t- test) (Fig. 1G). At investigation of tumor types separately, such an association was found only in SCC (282±75 G/l vs. 243±82 G/l, p = 0.007, t- test), but missed significance in AC (274±101 G/l vs. 247±73 G/l, p = 0.077, t-test). The presence of STC was associated with higher LMVD in all cases (16±7 vs. 12±5 microvessels/field; p<0.001, t-test) (Fig. 1H) and also in AC (16±6 vs. 12±5 microvessels/field, p<0.001, t-test) and SCC (17±7 vs. 13±6, p = 0.002, t-test) separately.

No direct association of STC or VTC with LVI was seen (p>0.05, Chi square test)., but in a linear regression model with LVI as dependent variable and including PBPC, STC and VTC showed that PBPC (p = 0.035, coefficient of regression<0.001) and VTC (p = 0.018, coefficient of regression 0,175) were associated with LVI.

In a second linear regression model using LMVD as dependent variable, including the same independent variables, again PBPC (p = 0.034, coefficient of regression −0.009) and STC (p<0.001, coefficient of regression 5.415) influenced LMVD.

### Survival Analysis

Mean observation time was 50±3 (standard error) months, during this observation period, 154 patients (48.1%) developed recurrent disease, and 131 (40.9%) died.

**Figure 2:**
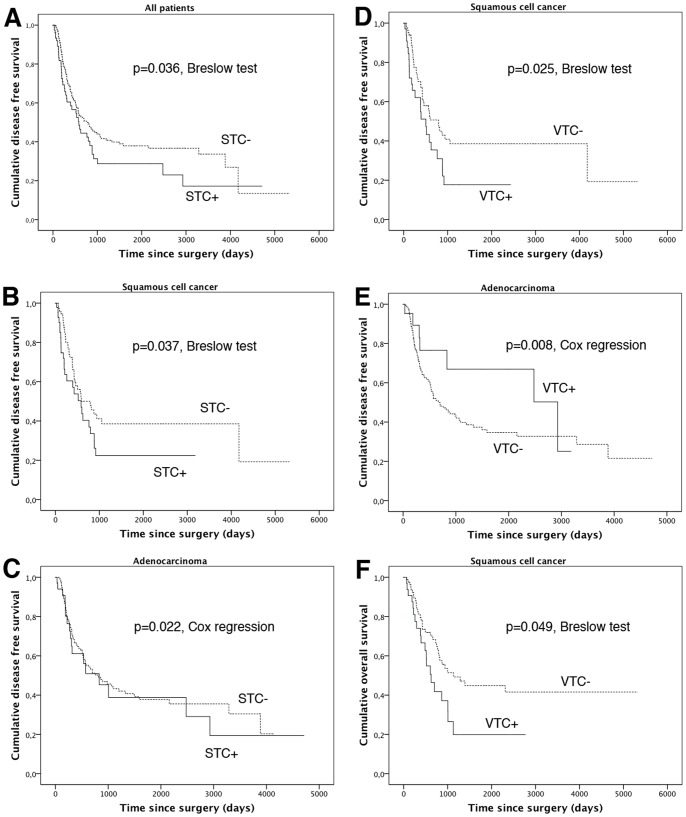
Kaplan Meier curves of disease free (DFS) and overall survival (OS) of esophageal cancer patients. Fig.2A: Presence of stromal thrombocytic clusters (STC) was associated with shorter DFS in all cases. At investigation of tumor types separately, STC was associated with shorter DFS in squamous cell cancer (SCC) (Fig. 2B) as well as in adenocarcinoma (AC) (Fig. 2C). Fig. 2D: Presence of vascular thrombocytic clusters (VTC) was associated with shorter DFS in SCC. Fig. 2E: Surprisingly, VTC was associated with significantly longer DFS in AC in multivariate analysis. Nevertheless, note that only relatively few events are seen in the VTC+ curve, and curves are crossing each other over, qualifying this finding. Fig. 2F: VTC was associated with shorter OS in SCC.

The presence of STC was associated with shorter DFS of all cases in univariate analysis (p = 0.036, Breslow test, Fig. 2A). At investigation of tumor types separately, no such influence was found in AC, but at analysis of SCC (p = 0.037, Breslow test, Fig. 2B). STC were associated with shorter DFS in multivariate analysis of AC (p = 0.022, Cox regression, table 2, Fig. 2C).

**Table 2:** Survival Analysis.

Factor	P-value univariate		P-value multivariate	Relative risk	95% CI
**Overall survival**					
**All tumors**					
STC	0.186		0.866	–	–
VTC	0.34		0.767	–	–
pT	<0.001		<0.001	1.713	1.332–2.202
pN	<0.001		<0.001	1.516	1.259–1.826
Grading	0.016		0.294	–	–
R0-resection	0.001		0.232	–	–
Patient age	0.129		0.189	–	–
Tumor type*	0.3		0.039	1.571	1.023–2.412
**Adenocarcinomas**					
STC	0.925		0.2	–	–
VTC	0.188		0.084	–	–
pT	<0.001		0.039	1.467	1.019–2.112
pN	<0.001		<0.001	1.64	1.266–2.124
Grading	0.003		0.523	–	–
R0-resection	0.005		0.499	–	–
Patient age	0.983		0.71	–	–
**Squamous cell cancers**					
STC	0.166		0.681	–	–
VTC	0.049		0.111	–	–
pT	0.003		0.002	1.867	1.265–2.754
pN	0.004		0.007	1.498	1.117–2.01
Grading	0.024		0.328	–	–
R0-resection	0.083		0.206	–	–
Patient age		0.998	0.971	–	–
**Disease free survival**					
**All tumors**					
STC	0.036		0.11	–	–
VTC	0.302		0.34	–	–
pT	<0.001		<0.001	1.73	1.374–2.177
pN	<0.001		<0.001	1.524	1.283–1.81
Grading	0.001		0.065	–	–
R0-resection	0.005		0.291	–	–
Patient age	0.982		0.042	0.983	0.968–0.999
Tumor type*	0.35		0.013	1.644	1.109–2.437
**Adenocarcinomas**					
STC	0.652		0.022	2.168	1.118–4.204
VTC	0.15		0.008	0.281	0.111–0.713
pT	<0.001		0.014	1.512	1.088–2.1
pN	<0.001		<0.001	1.679	1.325–2.127
Grading	<0.001		0.453	–	–
R0-resection	0.004		0.359	–	–
Patient age	0.979		0.018	0.975	0.955–0.996
**Squamous cell cancer**					
STC	0.037		0.669	–	–
VTC	0.025		0.401	–	–
pT	0.001		<0.001	2.014	1.378–2.944
pN	0.001		0.01	1.425	1.089–1.863
Grading	0.021		0.025	1.85	1.078–3.173
R0-resection	0.388		0.571	–	–
Patient age	0.991		0.279	–	–

univariate survival analysis of patients age was performed using univariate Cox regression.

* AC was associated with significantly better prognosis in multivariate analysis than SCC.

No influence of STC was seen on OS at investigation of all cases, as well at investigation of AC and SCC separately (p>0.05, Breslow test or Cox regression, respectively; table 2).

No relevance of VTC on DFS was seen at investigation of all cases. At investigation of AC and SCC separately, VTC was associated with shorter DFS in univariate analysis in SCC (p = 0.025, Breslow test, median DFS 506±82 vs. 794±139 days; Fig. 2D), but associated with longer DFS in multivariate analysis of AC (p = 0.008, Cox regression, median DFS 2928±990 vs. 700±141 days; Figure 2E). Presence of VTC was also associated with significantly shorter OS in SCC (p = 0.049, Breslow test, Fig. 2F).

PBPC was not associated with DFS or OS in uni-or multivariate analysis (p>0.05, uni- or multivariate Cox regression, respectively).

### Cell Culture

**Figure 3:**
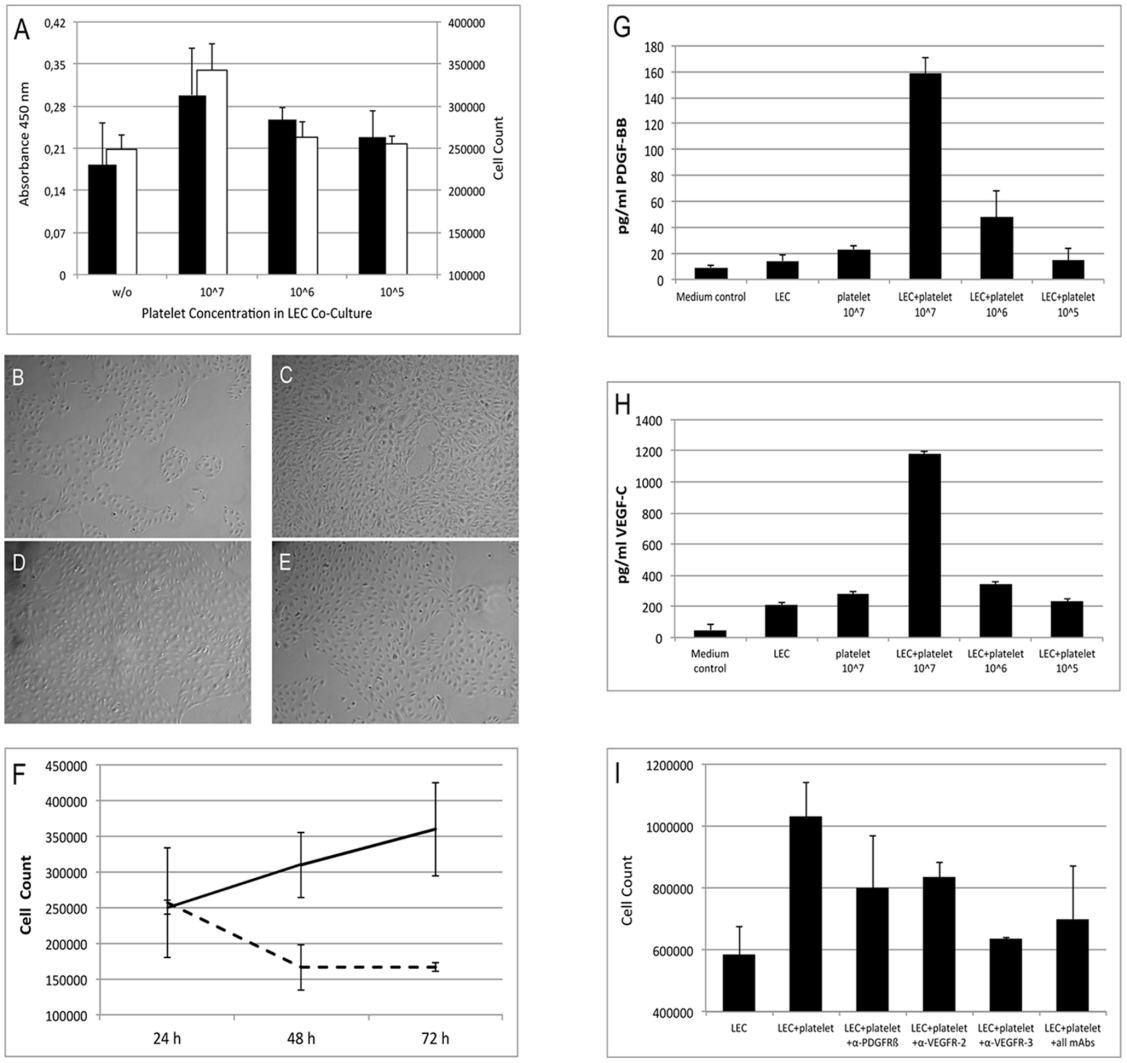
Cell culture experiments. A: LEC proliferation is enhanced by co-culture with human platelets in a dose-dependent manner. LECs were seeded at 1×10∧5 per 30 mm well. After 24 hours isolated platelets were added at 3×10∧7, 10∧6 or 10∧5 per well and cells were cultured for another 48 h. For quantification of cell proliferation, the LEC count was determined (black bars, right scale) and metabolic activity was measured by tetrazolium reduction assay (white bars, left scale). B–E: Corresponding microscopic images to A: B: Control; C: EC+Px10∧7, D: EC+Px10∧6, E: EC+Px10∧5. F: LEC proliferation is enhanced by co-culture with human platelets in a time-dependent manner. LECs were seeded at 1×10∧5 per 30 mm well. 24 hours thereafter isolated platelets were added at 1×10∧7 per well and cells were cultured for another 24, 48 and 72 hours. Cell counts were determined for LEC-platelet co-cultures (solid line) as compared to LECs without platelet addition (dashed line). G+H: Growth factor release during co-culture of LECs and human platelets. LECs were seeded at 1×10∧5 per 30 mm well. After 24 hours isolated platelets were added at 7×10∧7, 10∧6 or 10∧5 per well and cells were cultured for another 48 h. Culture supernatant was harvested, centrifuged (to remove cellular components) and then assayed for the concentration of PDGF-BB (G) and VEGF-C (H) by enzyme-linked immunosorbent assay. I: Platelet-induced LEC proliferation is mediated by PDGFRβ, VEGFR-2 and -3. LECs were seeded at 1×10∧5 per 30 mm well. After 24 hours isolated platelets were added at 7×10∧7 per well with or without blocking reagents against PDGFRß, VEGFR-2 and/or VEGFR-3. Cells were cultured for another 48 h before determining LEC counts.

LECs were seeded at 1×10∧5 per 30 mm well, and after 24 hours isolated platelets were added at 3×10∧7, 10∧6 or 10∧5 per well and cells were cultured for another 48 h. As shown in Figure 3A–E, LEC cell count increased with the number of added isolated platelets, indicating that LEC proliferation is enhanced by co-culture with human platelets in a dose-dependent manner.

As second experiment, we investigated if LEC proliferation is enhanced by human platelets in a time-dependent manner. For this purpose, platelets at 1×10∧7 per well were added to isolated LECs (1×10∧5 per 30 mm well) and cells were cultured for another 24, 48 and 72 hours. Fig. 3F shows that LEC proliferation is enhanced by co-culture with human platelets in a time-dependent manner compared to LECs without platelet addition.

As next step, we investigated if pro-lymphangiogenic factors are released during co-culture of LECs and platelets. Isolated platelets were added at 7×10∧7, 10∧6 or 10∧5 per well to LECs (1×10∧5 per 30 mm well) after 24 hours, and cells were cultured for another 48 h. Culture supernatant was harvested, centrifuged to remove cellular components and then assayed for the concentration of PDGF-BB and VEGF-C by enzyme-linked immunosorbent assay.

Figure 3G and 3H show that at a platelet concentration of 10∧6, PDGFR-β and VEGF-C were released, and this release of growth factors was strongly increased at a platelet concentration of 10∧7.

As a last step, blocking experiments were performed:

LECs were seeded at 1×10∧5 per 30 mm well. After 24 hours isolated platelets were added at 7×10∧7 per well with or without blocking reagents against PDGFRß, VEGFR-2 and/or VEGFR-3. Cells were cultured for another 48 h before determining LEC counts. Fig. 3I shows that LEC cell proliferation was in part reduced by the addition of the individual compounds in comparison to LEC/platelet co-culture without blocking substances. Inhibition of VEGFR-3 (blocking VEGF-C signaling) was most potent and decreased the platelet-mediated LEC proliferation by 90%. This effect could not be further enhanced by combination with anti-PDGFRß and anti-VEGFR-2 antibodies.

## Discussion

Platelets play an important role in human malignant disease: So it has been shown in many studies that preoperative elevated peripheral blood platelet count predicts poor prognosis in a variety of human cancers [17] among them also gastric cancer [18].

In esophageal cancer (SCC and AC), an increase in platelet count after en bloc resection has been reported to be associated with better prognosis in one study [19], and in a study from Pakistan, low preoperative platelet counts were associated with poor prognosis [20]. In contrast, another study found that preoperative thrombocytosis indicated poor prognosis in esophageal SCC [20].

Nevertheless, it is not completely clear if elevated platelet counts are induced by a more aggressive phenotype of tumors, or contribute themselves to clinical behavior of tumors. Most probably tumors may induce thrombopoesis directly e.g. via factor like IL-6 [21].

Platelets seem also to promote metastasis, but the exact mechanisms are still unclear [22], [23].

Platelets play also a role in tumor angiogenesis after activation, e.g. in response to endothelial damage [24]. This effect seems to be induced not only by platelet factor secretion, but also by direct stimulation of angiogenesis [25].

It is well known that peripheral human platelets are able to secrete pro-lymphangiogenic factors like VEGF-C and PDGFR-B, which are released during platelet activation [26]. In addition, platelets play an important role in the embryonic development of the lymphatic system [7]. So they have been reported to inhibit proliferation and migration of lymphatic endothelial cells during embryonic development upon activation by interaction of CLEC2 and podoplanin, which is expressed on lymphatic endothelial cells [27].

In malignant disease, the interaction between platelets and podoplanin via CLEC-2 induces a number of pathways involved in tumor cell migration and growth [28].

Surprisingly, to our knowledge no data on the role of platelets in lymphangiogenesis in human malignant disease do exist so far.

In our present study, we investigated in a large series of esophageal cancers the association of PBPC with platelets in tumor vessels, within the tumor stroma and with lymphangiogenesis.

Perioperative PBPC were associated with the presence of platelets within tumor tissue, and platelets within the tumor tissue was associated with increased lymphangiogenesis. While no direct association of STC or VTC with LVI of tumor cells was seen, PBPC and VTC influenced LVI in regression analysis.This indicates that a complex interaction between PBPC, VTC and STC promotes LVI of tumor cells.

To investigate our findings in detail *in vitro*, we performed cell culture experiments, showing that platelets promote growth of LECs in a dose- and time-dependent manner. This induction of LEC growth seems to be induced by secretion of pro-lymphangiogenic factors like VEGF-C and PDGF-BB.

So our findings might be a possible explanation why increased preoperative PBPC are associated with diminished prognosis in a variety of human cancers, although this was not evident in our study. Although podoplanin interacts with platelets during development, an interaction between lymphatic endothelial cells and platelets has not been described previously. This might be explained by the fact that thrombocytes are not found within the lymphatic vascular system. Nevertheless, platelets facilitate extravasation by the release of matrix metalloproteinases and by stimulation of tumor and endothelial cells [28]. As evident in our study, platelet clusters might be also found not only within blood vessels, but also within the tumor stroma, indicating leaking vessels. Since vascular and stromal platelet clusters correlated, the migration of platelets out of the vessels seems to be induced by vascular clusters. The lymphangiogenic factors secreted within the stroma by extravasated platelets might induce growth of lymphatic endothelium, thus supporting the formation of newly formed lymphatic vessels.

A shown in our cell culture experiments, this stimulation of proliferation of LECs by platelets seems to be induced in a time- and dose dependent manner mainly by VEGF-C and PDGF-BB, which are secreted by platelets. Blocking experiments indicate a predominant role of VEGF-C in this process.

As reported in a variety of studies, the increase in lymphatic vessels correlates with the probability to develop LVI and subsequent lymph node metastases. [29]–[34] The fact that platelets promote extravasation of tumor cells is well known [17], but based on our data it seems very probable that platelets in the tumor stroma also promote invasion of tumor cells into the lymphovascular system.

In summary, we show for the first time in large series of human cancer patients and also *in vitro* that peripheral blood platelets play an important role in esophageal cancer lymphangiogenesis and LVI, thus influencing prognosis of patients. So the disruption of signaling pathways between platelets, tumor cells and lymphatic endothelium might be of benefit for patients.
